# Training an AI Chatbot to Manage Health in Underserved Populations: Methodological Approach

**DOI:** 10.2196/84145

**Published:** 2026-04-01

**Authors:** Allison Diane Ihle, Breann Wicks, Vangelis Metsis, Autumn Starfall, Fleur Clapham, Aleksei Gorbachev, Sean Shanley, Christina Strauser, Jacqueline M McGrath

**Affiliations:** 1 The University of Texas Health at San Antonio San Antonio, TX United States; 2 Texas State University San Marcos, TX United States; 3 Scalable Care San Francisco, CA United States

**Keywords:** artificial intelligence, chatbot, digital health, mHealth, mobile health, women’s health, health disparities, criminal legal system, community supervision, pregnant, childbearing, parole, methods, women’s health, health

## Abstract

**Background:**

Health disparities such as morbidity and mortality among childbearing women remain high in the United States, especially among those with risks associated with criminal legal system involvement. These underserved women are often managed through community supervision such as probation. They have many needs and could benefit from easily accessible mobile health (mHealth) apps that specifically target their health and safety using artificial intelligence (AI).

**Objective:**

The purpose of this methodological case study is to provide our detailed strategies and findings for systematically designing, optimizing, and testing an AI chatbot.

**Methods:**

This methodological case study used an mHealth app’s AI chatbot, JUN, that involved preliminary studies and development efforts to support childbearing women on community supervision. We applied the Information Systems Research framework to guide the steps on how we designed, tailored, configured, and tested the chatbot using a retrieval-augmented generation framework. We demonstrated the feasibility of using an in-context learning approach addressing relevance, design, and rigor cycles.

**Results:**

During both crisis and noncrisis situations, the JUN chatbot had an overall performance of 89% accuracy (N=178) in detecting a “crisis.” Qualitative findings displayed increased usability of JUN to manage health at night by participants. The findings also demonstrated that the role of caregiving or current pregnancy was a motivating factor to manage health using technology such as the JUN app. Collectively, the sample expressed that barriers to managing their health effectively were associated with limited transportation, time off work, and insurance coverage. Participants in the community supervision group also described that stress related to criminal legal system involvement put limitations in how they managed their health and well-being. Altogether, participants from both groups discussed how an anonymous chat feature and app store accessibility would enhance the usability and acceptability of JUN among users. Pregnant women used the app to manage feelings of fatigue, shortness of breath, food cravings, anxiety, confidence, determination, frustration, excitement, happiness, hopefulness, irritation, love, as well as acknowledgment of their own feelings. Pregnant participants on community supervision had more housing (*P*=.05) and food (*P*=.01) insecurity, worry about electricity being turned off (*P*=.04), and needing resources (*P*=.01) compared to pregnant women without community supervision.

**Conclusions:**

We illustrate the methodological case study to design, optimize, and test an AI chatbot within an mHealth app to provide health and safety-related support for childbearing women on community supervision. This methodological case study poses possibilities for further development and testing of interventions for populations with similar risks to their health and safety.

**Trial Registration:**

ClinicalTrials.gov NCT06636110; https://clinicaltrials.gov/ct2/show/NCT06636110

## Introduction

### Theory

Mobile health (mHealth) apps, integrated within smartphones, have the potential to be supportive of health care services [1]. A sizeable number of individuals within the United States—approximately 98%—have access to a cellphone [2]. In fact, since 2011, smartphone ownership has risen 56%, with 9 in 10 (91%) American people owning smartphones [2]. Consequently, smartphone technology has become integrated into the daily lives of people across the nation, with users sending and receiving an average of 6 billion SMS text messages per day or roughly 70,000 SMS text messages every second [1]. Because of this prevalence, the use of smartphone apps can be considered a strategy for improving individual and community health.

Smartphones are poised to transform health care management to become more efficacious for preventative methods, early detection, diagnosis, and treatment or management of a range of health care conditions [3]. The use of mHealth apps with longitudinal monitoring and data collection of physiological parameters illustrates functionality that enables health behavior and symptom tracking, access to personal or population-based health data, medication adherence, and 2-way communication between the user and their health care provider [4]. Moreover, serving as a 2-way interactive communication tool, mHealth apps can present opportunities for health care providers, in collaboration with researchers and program developers, to leverage existing cultural behaviors of underserved populations [1].

### Background

Advances in mHealth apps have helped health system interventions shift from the physician-led clinic model historically used to deliver health care services to a tailored model of patient-centered care, which will likely allow sustained monitoring and improve engagement, usability, and acceptability among underserved populations [5-7]. Therefore, mHealth apps specifically used among underserved populations must be designed with inclusivity using validated tools to promote engagement, retention, trust, and overall acceptability and usability [8]. Further, enhancing mHealth app functionality with artificial intelligence (AI) chatbots holds great promise for improving patient outcomes and system efficiency, particularly accessing predictive modeling and augmenting rather than replacing clinical-led decision-making [7,9].

Successful user engagement must incorporate client choices, preferences, and adaptive content to embrace cultural diversity and user behavior [5,10]. In support of this notion, mHealth tools that use AI chatbots have been adapted to access large language models (LLMs) [9,11]. This method can introduce specific cultural norms, using region-specific language and nuanced community values [9,12,13]. Thus, LLMs can leverage natural language processing to provide refined, adaptive, human-like communication and understand multifarious queries with precision while producing detailed, context-specific responses [11,12,14]. However, the majority of LLM-based AI mHealth apps offer broad guidance regarding general health concerns unless users manually tell the app their health information, limiting the overall effectiveness and precision in managing complex conditions [11]. LLMs that use plain language, which has been written or reviewed by populations of interest, may mitigate stigma, increase health access, and build patient trust [6,11].

### Hypothesis

Underserved populations often have limited contact with health care providers and low engagement with managing their health due to socioeconomic constraints [6]. Health care barriers include limited access to health insurance, high cost of medical appointments, limited transportation or childcare, and fear of discrimination, making traditional health care methods, such as face-to-face in-office appointments, inaccessible [6]. At the same time, smartphone technology has also become the primary source for internet access for populations of lower socioeconomic status and education level [2]. Hence, interventions that use smartphone technology may be a cost-effective and convenient way to engage hard-to-reach underserved populations with health care services.

### Framework

The Information Systems Research (ISR) framework has been used in prior research [15] when designing mHealth apps, which applies several processes within the development, implementation, and testing of an mHealth app [16]. The ISR framework consisted of 3 phases, which can take place iteratively versus linearly. The first phase is the relevance cycle, in which data are collected to better understand the environment of the end user by a series of interactions with the population of interest and stakeholders through interviews. The second phase is the design cycle, in which output from the app is produced and evaluated. The third phase is the rigor cycle. During this phase, end-user perspectives and output are evaluated in relation to design science and app domains [16].

The purpose of this methodological case study is to outline in detail our strategies and findings in relation to designing, tailoring, testing, and training an AI chatbot within an mHealth app using the ISR framework, relevance, design, and rigor cycles. It is important to remember that these phases are not linear, and the work in each phase overlaps with other phases. We provide a methodological case study approach demonstrating how we executed each phase among a population of interest, childbearing women with histories of criminal legal system involvement, such as community supervision.

## Methods

### Ethical Considerations

This study was reviewed and approved by the University of Texas at San Antonio’s Health Science Center’s Institutional Review Board (IRB; STUDY00001062). All participants provided informed consent prior to participation. The study was conducted in accordance with the Declaration of Helsinki and all applicable institutional and federal guidelines. This study did not involve participant incentives. Data were deidentified prior to analysis, stored on secure, password-protected servers, and accessed only by authorized study personnel.

### Case Study: Childbearing Women With Histories of Criminal Legal System Involvement

Genetic and environmental causes of maternal morbidity and mortality affect roughly 50,000 women each year in the United States [17]. For every woman in the United States who dies from pregnancy, 20 to 30 more women experience lifelong complications that impact their well-being and health, contributing to the highest maternal mortality rate compared to any other country [17], with every 3 in 5 pregnancy-related deaths being preventable [18]. Socioeconomic factors, including lower education levels, lack of insurance or underinsurance, low health literacy, inessential cesarean delivery, chronic comorbidities and complications, as well as issues related to pregnancy and childbirth, contribute to increased maternal morbidity and mortality among women of childbearing age (ages 18-50 years) in the United States [17,19,20]. These factors heighten risks for individuals younger than 24 years or older than 35 years, with race and ethnicity serving as risk markers [17].

Childbearing women with histories of criminal legal system involvement raise risk factors that contribute to maternal morbidity and mortality [2,20]. The rates of morbidity and mortality increase after initial arrest and interaction with the criminal legal system and sustain while women are released back into the community [21,22]. Women of childbearing age are often prioritized for community supervision, also known as probation, which is a type of criminal legal system condition that is an alternative to being detained in jail or prison to complete a criminal sentence [23,24]. Despite community supervision being less restrictive than jail or prison, the circumstances of this type of oversight enhance socioeconomic barriers for this population of women [25-27]. Hence, most of these women are considered hard-to-reach, often have low rates of interacting with health care providers, and limited self-management of their health [28-30].

### Methodological Approach

By applying the ISR framework and using a case study design that uses qualitative and quantitative analysis, we first discussed the methodological approach to designing, implementing, and testing an mHealth AI chatbot by addressing (1) the relevance cycle, (2) the design cycle, and (3) the rigor cycle outlined in the ISR framework [16].

### Dataset Development and Corpus Construction

Recruitment and data collection across qualitative and pilot studies within the relevance and rigor cycles were between 2020 and 2025. Across studies, 82 childbearing women (ages 18-50 years) with histories of criminal legal system involvement (n=57) or who were pregnant and without criminal legal system involvement (n=25) were recruited. A review of the literature was conducted in 2022 for 231 papers specific to mHealth apps within criminal legal system spaces.

To enable context-aware responses without fine-tuning, we developed a comprehensive retrieval corpus comprising 5738 unique natural language utterances (after removing duplicates from a total of 6461 inputs). These utterances span 29 distinct domains relevant to the target population, including mental health, safety, and criminal legal system challenges. The average utterance length is 6.25 (SD 2.3) words, reflecting the concise, conversational style of the target population. Lexical diversity analysis revealed a vocabulary of 4225 unique words (type-token ratio=0.12). The examples covered the category and challenge domains (Table 1).

**Table 1:** Categories and challenges within JUN that were used to inform the algorithm.

Domain type	Domain names
Categories	Caregiving Relational health General health Reproductive health Trauma Mental health Nutrition Safety Identity
Challenges	Mental health Criminal legal system involvement Parenting Caregiving Violence Abuse Substance use disorder Grief Major life change Loss of autonomy Family separation Isolation Pregnancy Childbirth Postpartum General health Ability Aging Identity Loss

In January 2025, the initial sentences were created by 6 different individuals specific to each of the 9 category and 15 challenge domains (Table 1) to total 2400 sentences (category sentences: n=900 and challenge sentences: n=1500). Individuals identified as male (n=1) or female (n=5) and were between the ages of 18 and 40 years. One member had their PhD, another had their masters, and 4 were undergraduate students. The team comprised of 2 subject experts specific to issues involving women and the criminal legal system. The team was instructed to create sentences in natural language using preliminary qualitative data that were informed from childbearing women within criminal legal system spaces.

Individuals were instructed (1) to create sentences in language-like texting; (2) to reframe from using ChatGPT; (3) to apply personal, professional, or contextually relevant scenarios; and (4) to use complete or incomplete sentences, slang, culturally relevant jargon, and/or misspelled words. Then, in March 2025, the second training session was initiated, with participants adding 120 more sentences in natural language specific to each category and challenge (Table 1) to total 2880 sentences (category sentences: n=1080 and challenge sentences: n=1800) to retrain, tailor, and test the JUN AI chatbot. Therefore, altogether, there were a total of 5280 natural language sentences created by the sample (n=6) to conduct 2 different training sessions of the JUN AI chatbot.

## Results

### Overview

**Figure 1:**
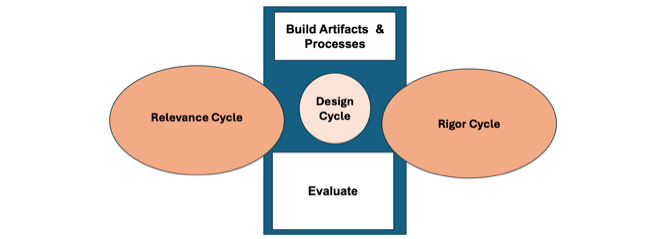
Information Systems Research framework.

Using a case study approach and the ISR framework, we discuss strategies for designing and testing an mHealth app, called JUN, which uses AI chatbot technology specific to three different phases: (1) relevance, (2) design, and (3) rigor (Figure 1) [15].

### Phase 1: The Relevance Cycle

#### Overview

The first phase of the ISR framework is the relevance cycle, in which data are collected to better understand the environment of the end user through a series of interactions with the population of interest and stakeholders [16]. The best approach to enhance usability, acceptability, and cultural sensitivity of mHealth apps among underserved groups is to apply an intersectional approach that considers the positionality of users and centralizes their preferences from the beginning stages through development and tailoring [1,6,31,32]. The social, economic, and political barriers among women within the criminal legal system and factors that reduce self-efficacy or the way women effectively manage their health have been documented [25,26,28,30,33,34].

However, limited data exist regarding the experiences and needs specific to Hispanic women in South-Central Texas, where this research originated. Additionally, data that focus on implementation science in community supervision spaces [35,36], the health outcomes of women on community supervision [37], and mHealth apps to assist women with histories of criminal legal involvement in managing their health are limited [38]. Therefore, the preliminary steps to designing the mHealth app, JUN, were undertaken by conducting qualitative interviews to understand (1) the experiences [39]; (2) the gaps and extent of the literature specific to smartphone app technology; and (3) the feasibility, usability, and acceptability of mHealth apps [40] among childbearing women involved with the criminal legal system.

#### Step 1: Exploring the Experiences of Women

In 2020, the first preliminary study was conducted using a descriptive qualitative design [41,42] and the reproductive justice framework [43] to organize results. This study provided insight into the experiences of 12 women of childbearing age (18-50 years) who self-identify as Latina who had histories with the criminal legal system [44]. We used semistructured, individual interviews to address the research question, “What are the experiences of Latina mothers impacted by incarceration?”.

We applied the reproductive justice framework to inductively sensitize the experiences with respect to the right to bodily autonomy, right to have or not have children, and right to parent in safe and sustainable environments following arrest [39,45]. An overarching theme (We are still human) and 4 major themes (I did whatever they wanted me to do, It is me against the world, Even through the pain you push through, and Our voices have not been heard loud enough) emerged from the data [39,45]. Themes described experiences of discrimination, trauma, barriers, resiliency, and desires, following arrest.

This study highlighted the socioeconomic barriers of these women who are associated with the criminal legal system at the intersection of being pregnant, birthing, and parenting children, as well as the egregious instances of coercion, abuse of power, stigma, and sexual violence [39,45]. The findings indicated a need for more research and tailored interventions that bridge access to health care and community services and that take an intersectional approach that considers women’s unique needs based on their gender, position within the criminal legal system, and roles as caregivers [39,45].

Findings also highlighted how ineffective coping and limited self-management of one’s health can be overshadowed by the demands of community supervision while being a mother to young children [46]. This study took place during social distancing due to the COVID-19 pandemic; hence, data collection was completed virtually over the telephone. This data collection method provided a foundation for considering how digital technology could be a convenient and discrete method for engaging with this population. Therefore, implications for the future direction of research were directed at exploring how technology can bridge access to health care for this hard-to-reach population of women. Details of the interview guide and demographics can be found in Tables S1 and S2 in Multimedia Appendix 1.

#### Step 2: Identify Gaps in Existing Evidence

**Figure 2:**
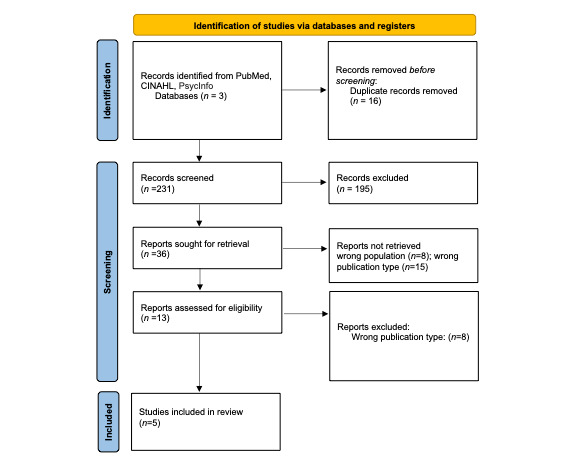
PRISMA (Preferred Reporting Items for Systematic Reviews and Meta-Analyses) diagram of findings.

In 2022, building on our previous work, a scoping review was conducted to examine the existing literature specific to research with mHealth apps to address the health needs of women with criminal legal system involvement [38]. We searched CINAHL, PubMed, and PsycInfo for peer-reviewed research papers conducted in the United States [38]. After removal of duplicates, review of 231 paper titles and abstracts and 36 papers for full-text review yielded 5 papers for inclusion [38]. Only 1 study was specific to women on community supervision [38]. Of the 5 studies that addressed health disparities of individuals under community supervision, 1 was completed with participants on community supervision, 3 with participants on medication therapy for substance use disorder, and 1 with participants in a court-mandated drug court program [38]. In addition, none of the studies regarding mHealth apps used an intersectional lens and qualitative design to tailor and develop the technological intervention; therefore, this gap in study design further justified the urgency to develop an mHealth app specific to the needs of this population of women (Figure 2) [38,47]. Details of the data extraction such as the study design, sample, objectives, conclusions, measures, intervention, analytic approach and PRISMA (Preferred Reporting Items for Systematic Reviews and Meta-Analyses) diagram can be found in Tables S1 and S2 in Multimedia Appendix 2.

#### Step 3: Intervention Development Study to Test the Feasibility, Usability, and Acceptability of mHealth Apps

Finally, in the fall of 2023, a third exploratory pilot study using qualitative and quantitative methods was conducted among 11 women who were of childbearing age (18-50 years) with histories of community supervision. We piloted 2 existing apps with topics closely related to women’s needs on community supervision. Participants were instructed to interact with 2 existing mHealth apps that were originally designed to manage sexual health (Nice Intimacy Tracker [48]) and dating violence (USafeUS [49,50]) among college women [40]. Neither app had AI functionality, but rather were designed to be more educational in nature. Individual telephone interviews guided by the social cognitive theory were used to gather information for data synthesis and organization to understand health behavior using both mHealth apps and what participants liked and disliked about both apps to inform the development of JUN. In addition, descriptive statistics were used to examine the usability of both apps using the mHealth App Usability Questionnaire. Three themes emerged: (1) It (the app) made me take time for myself, (2) It (the app) helped me to be more respectful of my body, and (3) The connectivity ... that was helpful.

Findings also highlighted that women on community supervision were open to using mHealth apps to manage their health. Participants voiced that mHealth app use allowed them to understand their behavioral patterns to manage their health more effectively, providing them with insight into the trends over time of potentially harmful health practices. As such, results illustrated that mHealth apps addressed the environmental barriers related to being caregivers while having criminal legal system involvement, potentially streamlining health management for underserved women. Finally, this research suggested that a culturally tailored mHealth app that uses an AI chatbot may be an appropriate intervention to provide timely gender-responsive feedback, resources, and health care to women on community supervision. Details of the mHealth app’s functions that were piloted, the interview guide, and findings related to experiences of violence and reporting are in Tables S1-S3 in Multimedia Appendix 3.

### Phase 2: The Design Cycle

#### Overview

The second phase of the ISR framework is the design cycle, in which app output is produced and evaluated [16]. The findings from all 3 preliminary studies informed the need to begin the development of an mHealth app, JUN, which was created to address the health and safety-related needs of women with criminal legal system oversight. Initially, the development, sustainment, and management of JUN was university-driven using intramural research funding awarded by the University of Texas at San Antonio Health Science Center. ADI (principal investigator) from the University of Texas at San Antonio Health Science Center led the preliminary studies that informed the artistic, conceptual, and functional design, while a student collaborator within the Computer Science Department developed the first JUN prototype.

#### Proof of Concept

JUN’s prototype was developed between November 2023 and June 2024 by an interdisciplinary team and partnership between the computer science and nursing departments. The first iteration of the JUN app was a proof-of-concept that did not have AI ability, which was used to communicate the overall concept, use, and basic functionality of the app. Features of the first prototype did not have 2-way interaction and were more based on user-guided interaction and input with imagery, such as the JUN logo and color scheme, illustrating that the initial artistic palette design was portrayed in the prototype.

In June 2024, administrative management concerns regarding maintenance, support, sustainability, scalability, and cost arose; therefore, in August 2024, a university-vendor collaboration with Scalable Care was formed. Scalable Care is a start-up technology company based in San Francisco, which took over the management and development of JUN. This partnership was essential in further developing and scaling the intervention to have the ability to execute larger clinical trials and more sophisticated in-app functionality. Following the university-vendor partnership, the design process began, which is comprised of three steps: (1) the artistic design, (2) the conceptual design, and (3) the AI chatbot design.

#### Step 1: The Artistic Design

**Figure 3:**
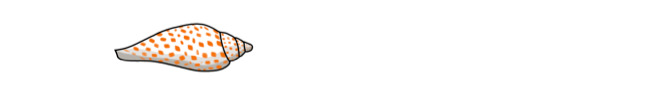
The junonia shell.

**Figure 4:**
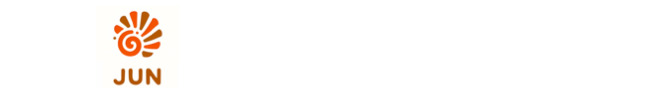
JUN logo.

JUN is short for the junonia shell (Figure 3), a native shell of South Texas, which means courage, strength, and self-sufficiency, much like the women who informed its design [39,40]. In addition, the orange color of the native shell is symbolic of the institutions where this research originated (The University of Texas System). Therefore, the name JUN was contextually relevant to be a discreet, meaningful, and symbolic name for the mHealth app and its logo (Figure 4), which was trademarked in 2025.

**Figure 5:**
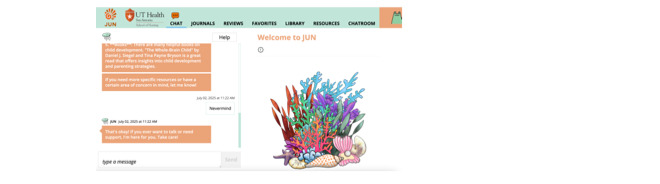
JUN wireframe.

**Figure 6:**
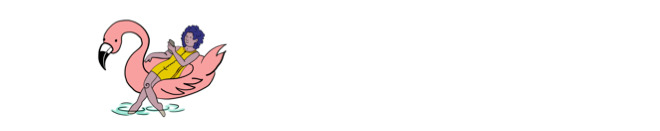
Images of artistic graphics in JUN.

Another area that was included in the artistic design process was the color scheme, feel, and imagery within JUN. Working with an art faculty member from the University of Texas at San Antonio, the colors were tested and selected for the app using a chromatic circle wheel and applied in areas around the border and tabs of the app (Figure 5). In addition, collaboration took place to develop, refine, and tailor images that would be fitting, representative, and inclusive for end users; the design prioritized different body types and used fictional hair and skin tones that are playful in nature (Figure 6).

#### Step 2: The Conceptual Design

JUN applies a conversational AI chatbot powered by LLM technology, including ChatGPT, to provide health-related support, feedback, education, and guidance across health, safety, and social domains. The app is available in 8 languages that are dominant within the United States and allows the customization of the user avatar to enhance the user experience. Table 2 displays the functionality within JUN that consists of features informed by the target population in preliminary studies [39,40].

**Table 2:** The conceptual design related to the functions within JUN.

Function name	Function meaning
1. Chat	A conversation feature to interact and get advice from the JUN AI^a^ chatbot
2. Safety	Safety features that allow fake calls and texts from the AI chatbot to get out of a harmful situation
3. Library	An education tab with trauma-informed evidence-based nursing education around general health, reproductive and sexual health, mental health, substance use, caregiving, criminal legal system involvement, and relational health
4. Journal	A symptom tracker feature that gives statistics and trends and serves as a virtual AI-led triage function that is trained using nursing rationale that gives feedback regarding health, safety, and social-related symptoms
5. Chatroom	An anonymous chat that allows in-app users to interact with each other through online forums or posts
6. Resources	An AI-led resource tab that intuitively gives resources specific to the user’s needs

^a^AI: artificial intelligence.

#### Step 3: The AI Chatbot Design

The health, safety, and social-related domains or features within the mHealth app, JUN, have been trained around categories and challenges that were informed by preliminary data specific to the needs of women with criminal legal system involvement. Table 1 displays all 9 categories and 20 challenges used in training the JUN algorithm. Next, we discussed the methods used to apply these categories and challenges to develop the JUN AI chatbot’s algorithm to be sensitive in giving context-specific feedback regarding the health, safety, and social concerns of childbearing women with experiences of criminal legal system involvement.

#### LLM to Inform AI Chatbot Design

Recent methodological approaches to training AI chatbots using natural language have leveraged advances in LLMs, such as ChatGPT, to improve task-specific performance in the health domain [51]. However, the deployment of AI chatbots in health-related applications requires a high degree of rigor, particularly in scenarios where users are experiencing acute and complex health-related issues, such as a mental health crisis or thoughts of suicide. Hence, there is an urgent need for relevant, domain-specific training data balanced with the ethical challenges of collecting time-sensitive mental health conversations [52].

For any such model, the output of an AI chatbot depends on its large and often unknowable set of training data [53]. For instance, training the bot around sensitive issues such as a mental health crisis (ie, thoughts of suicide or harming others) is essential to adequately address the needs of populations with risks associated with criminal legal system involvement [52]. Numerous commercial chatbots, such as Character.ai, a virtual therapist site with over 20 million chat interactions captured, have recently been reported to violate company policy related to child safety, user data, and content moderation [54]. These crisis detection dialogues were not removed due to their popularity and highlight the need for more rigorous and therapeutically informed methodologies when training and deploying AI chatbots in mental health care [54].

#### Contextually Tailored LLM to Inform AI Chatbot Design

In contrast, specific AI chatbot platforms such as Woebot were developed for specific user populations using their own cognitive behavioral therapy frameworks or philosophy in how they treat and manage individuals with various mental and physical conditions [54,55]. Comparable to the process developed for JUN by Scalable Care, these AI chatbots use curated language patterns to deliver supportive, empathetic conversations [54,55]. However, despite LLM’s ability to potentially achieve diagnostic accuracy, the natural language responses may fall short of therapeutic standards [56].

These LLM models may capture stigmatizing content when certain populations engage in behaviors such as substance use, highlighting the need for ethically grounded, strict oversight of the data and output [57]. Therefore, users of the target population must provide feedback on trained AI chatbots around different cultural, health, behavioral, and socioeconomic domains to ensure that the emotional tone and linguistic style are in alignment with users’ expectations [58]. Hence, the methods we have used facilitate a context-sensitive, user-informed approach in training AI chatbots geared for health-related settings that involve training the algorithm using preliminary data from qualitative interviews and user feedback following pilot-testing to ensure that the app responds in a culturally sensitive manner.

### Phase 3: The Rigor Cycle

#### Overview

The third phase of the ISR framework is the rigor cycle. This process involved evaluating JUN’s output combined with the perspectives of end users [16].

#### Generative AI Architecture

The JUN chatbot uses a retrieval-augmented generation framework [59] combined with dynamic few-shot prompting [60]. Rather than fine-tuning a model’s weight, the system uses a 2-step pipeline using OpenAI models (GPT-4o-mini) [61]:

- Retrieval: When a user inputs text, the system calculates the semantic similarity between the input and the 5738 examples in the retrieval corpus.
- In-context learning: The most relevant examples, both positive matches and negative “fall-back” examples, are dynamically retrieved and inserted into the system prompt. This allows the LLM to classify the user’s intent (eg, “crisis” vs “general health”) and generate a response that mimics the empathetic, safety-conscious tone of the retrieved examples.

#### Fallback Mechanism

If the LLM confidence score falls below a set threshold, the system triggers a fallback protocol to ensure that the user receives a supportive, noncommittal response rather than a hallucination. The first step the team took in executing our approach was to first identify what the categories and challenges were (Table 1) based on preliminary data and user feedback [39,40]. We began the first training session of the AI chatbot with 2400 items written in natural language (Table 1). Then, we conducted a second training of 2880 items in natural language to retrain, tailor, and test the JUN AI chatbot and run pre- and postreliability and validity testing, resulting in 5280 items total.

During both steps following the creation of the sentences in natural language, the research and engineering team reviewed and approved training sentences that were balanced across the different categories and challenges (Table 1). This process included defining intent classifiers, interpreting training data, engineering prompts, and rigorously validating the system using a quality assurance workflow. This approach has been designed to maintain both safety and domain relevance responses to concerns raised about unpredictable and potentially harmful outputs of LLMs in health contexts [53]. Thus, classifiers provide consistent, interpretable, and domain-controlled outputs, while LLMs augment user experience through conversational flexibility [53]. This fused architecture, whereby classifiers govern core functionality, and LLMs provide naturalistic dialogue, illustrates a compelling, scalable, and adaptive model for the effective deployment of intelligent virtual assistants in health-related mHealth apps [53].

#### Testing the Rigor of the AI Chatbot

We used OpenAI’s GPT-4o-mini [61] for intent classification and response generation within the app. Conducting qualitative interviews to gauge the perspectives of users’ experiences to guide the future direction of app development is essential. Furthermore, conducting validity and reliability by running pre- and posttests of the AI chatbot’s algorithm is essential in evaluating the effectiveness of the intervention [6,16,62]. Currently, several tests have been conducted.

#### Rigor Cycle Study 1

##### Overview

The first study was a pilot study testing JUN’s ability to enhance self-efficacy among pregnant women, comparing (n=25) women who are on community supervision to those (n=25) without criminal legal system involvement. Participants had to be pregnant and under 6-month gestation, have a smartphone with internet, and could read and write English. The frequency of app use, behavioral patterns, and outcomes of participant (n=50) interactions with JUN were retrieved. Qualitative data guided by the health belief model [63] and 2-tailed *t* tests were conducted to examine quantitative data related to differences in self-efficacy and app usability data between groups regarding their pregnancy-related outcomes and health behavior across 3 months.

##### Findings

Details of the interview guide can be found in Table S1 in Multimedia Appendix 4. Qualitative findings displayed increased usability of JUN to manage health at night by participants. The findings also demonstrated that the role of caregiving or current pregnancy was a motivating factor to manage health using technology such as the JUN app. Collectively, the sample expressed that barriers to managing their health effectively were associated with limited transportation, time off work, and insurance coverage. Participants in the community supervision group also described that stress related to criminal legal system involvement put limitations in how they managed their health and well-being. Altogether, participants from both groups discussed how an anonymous chat feature and app store accessibility would enhance the usability and acceptability of JUN among users [Ihle, unpublished data, 2026]. Usability quantitative findings included the use of JUN more on Mondays and Wednesdays across the sample [Ihle, unpublished data, 2026]. Pregnant women had feelings of fatigue, food cravings, anxiety, confidence, determination, frustration, excitement, happiness, hopefulness, irritation, love, acknowledgment of feelings, communicating clearly, giving options, time and space, and shortness of breath [Ihle, unpublished data, 2026]. When comparing pregnant women on community supervision to pregnant women without criminal legal system involvement, women on community supervision had more housing (*P*=.05) and food (*P*=.01) insecurity, were worried about their electricity being turned off (*P*=.04), and needed help finding resources (*P*=.01) [Ihle, unpublished data, 2026]. Results that were trending toward significance among women on community supervision were having newborns with health complications (*P*=.08), past complications in childbirth (*P*=.08), complications with the current pregnancy (*P*=.08), anxiety during the current pregnancy (*P*=.06), and not being able to stand up for rights effectively (*P*=.07), trending toward significance. Given the exploratory nature of this pilot study, these *P* values should be interpreted as descriptive signals of potential disparities rather than confirmatory hypothesis testing.

#### Rigor Cycle Study 2

##### Overview

The second study, exploring JUN, conducted in 2025, used a qualitative design and thematic content analysis guided by the social cognitive theory [64,65] to explore the perspectives of childbearing women who had histories of community supervision on how they manage their health. In total, 10 individuals who self-identified as women between the ages of 28 and 50 years were sampled. Inclusion criteria were that they had experience with community supervision, owned a smartphone with internet, and could read and write in English. Questions regarding the usability, acceptability, and feasibility of JUN were asked to participants. Using qualitative thematic content analysis, findings were analyzed around the tenets of the social cognitive theory [64,65] such as (1) personal factors, (2) behavioral factors, and (3) environmental factors. Participants were instructed to use JUN daily as needed across 1 week. Qualitative interviews were then conducted over the telephone. The interview guide is in Table S1 in Multimedia Appendix 5. One person failed to be recontacted; therefore, only 9 individuals were interviewed. Recruitment and data collection stopped at the ninth interview after data saturation.

##### Findings

Results specific to (1) personal factors included usability at night within the home [Ihle, unpublished data, 2026]. Most participants disclosed being busy as caregivers, making use during the day or at work was difficult [Ihle, unpublished data, 2026]. Participants enjoyed using the app to manage their health and were open to using the app despite not having used technology to manage their health prior to participation [Ihle, unpublished data, 2026]. (2) Behavioral factors included liking features such as the symptom tracker, AI reminder prompts, and education tab [Ihle, unpublished data, 2026]. Some users also described liking the audio feature to listen to content while driving [Ihle, unpublished data, 2026]. Functionality suggested by participants was an anonymous chat among in-app users, accessibility to download JUN on the App Store and linking JUN to real health care providers [Ihle, unpublished data, 2026]. (3) Finally, environmental factors included using JUN more at home. Participants disclosed using the app in the community or work setting was difficult due to time and confidentiality issues [Ihle, unpublished data, 2026].

#### Rigor Cycle Study 3

**Figure 7:**
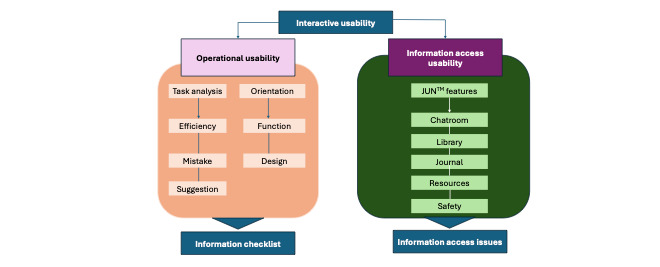
Usability evaluation model.

**Figure 8:**
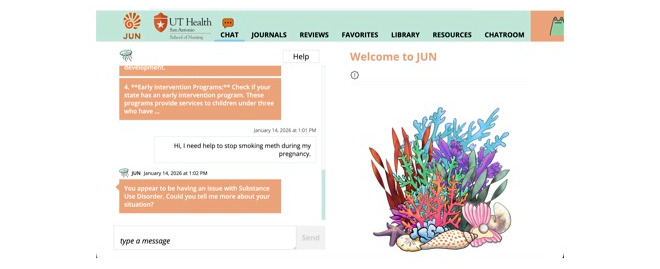
Wireframe of the JUN mobile health site demonstrating the JUN chatbot dialogue feature.

Finally, the third study tested the validity, reliability, cultural sensitivity, and usability of JUN’s AI chatbot algorithm. This study used data informed or extracted from qualitative excerpts of preliminary studies [39,40,45,46] to inform the natural language sentences developed by the research team around the categories and challenges (Table 1). A preintervention interview using a structured interview guided around the category and challenge domains (Table S1 in Multimedia Appendix 6) was conducted, transcribed, and uploaded into Dedoose—an online program to assist with qualitative analysis (SocioCultural Research Consultants, LLC). The research team member who interviewed the JUN chatbot was instructed to go through each category and topic using the same sentences as in the interview guide and allow time for the chatbot to respond. After all questions were asked, they were then instructed to state “never mind” in the chat to redirect the JUN chatbot to another topic. Interview results along with the output given by JUN were analyzed using the usability evaluation model (Figure 7) [66] within Dedoose (Figure 8).

#### Crisis Detection Performance

To evaluate the safety of the system, we conducted a performance test on a dataset of 178 utterances (crisis: n=104 and noncrisis: n=74). The “crisis” examples included high-risk inputs (eg, “I want to end it all”) verified by the principal investigator (ADI). The “noncrisis” examples included a mix of general health queries and synthetic inputs generated to test the model’s ability to distinguish between distress and casual conversation.

On this held-out test set, the system achieved an overall accuracy of 89% (Table 3). The system demonstrated high precision (0.98) for the crisis category, indicating a low rate of false alarms. However, the recall for “crisis” events was 0.83, suggesting that while overt threats are caught, some ambiguous distress markers may require further sensitivity tuning.

**Table 3:** Testing the definition of a “crisis.”

	Precision	Recall	*F*_1_-score	Support
CRISIS	0.98	0.83	0.90	104
NOT_CRISIS	0.80	0.97	0.88	74
Overall or average	0.89	0.89	0.89	178

Qualitative findings included similar feedback from the JUN AI chatbot during pre- and posttraining around the educational content [Ihle, unpublished data, 2026]. However, the posttraining interview had more suggestions by the JUN chatbot specific to community resources [Ihle, unpublished data, 2026]. In addition, the pretraining interview had more errors and suggestions that had not completely addressed all concerns from the participant [Ihle, unpublished data, 2026]. In comparison, the posttraining interview had the chatbot conversing with the participant more and taking them through resources within the app such as the education, resource, and symptom tracker features while also suggesting community resources outside of the app [Ihle, unpublished data, 2026]. Conclusively, the results from these 3 studies within the rigor cycle have informed the validity, reliability, usability, and cultural sensitivity of the JUN AI chatbot, strengthening assumptions and guiding future tailoring and sustainment.

## Discussion

### Overview

mHealth apps have gained recognition in mitigating the health-related needs in groups that are considered hard-to-reach, have risks to their social determinants, and have low self-efficacy [1,5,6,8]. Populations with heightened health risks that are hard to reach could greatly benefit from mHealth apps that use an AI chatbot. This technology can bridge access to care and give contextually relevant health, safety, and social support.

### Principal Findings

#### Overview

The systematic methodological case study we used to develop, tailor, and test JUN, an mHealth app that uses an AI chatbot, may be a blueprint for other scholars to replicate to develop interventions for other populations to enhance health outcomes. However, there are some factors to consider when applying our methods toward other populations such as (1) required resources and expertise, (2) exploratory preliminary pilot studies, (3) geographic location, (4) methodological aspects that may be transferable or context-specific, (5) cost estimates for development and maintenance, and (6) scalability challenges.

#### Required Resources and Expertise

Interdisciplinary collaboration is essential. The team informing intervention development must have a subject matter expert who knows the data phenomenon, and the population of interest is necessary to be able to guide and oversee development across all stages. Other expertise necessary are computer engineers, specifically those with expertise in AI, LLM, and ethical-related concerns regarding protection of human subjects; statisticians who have expertise in analysis using large datasets within wearable devices to predict human behavior such as ecological momentary assessment; legal counsel on forming Business Associate Agreements detailing proposer use, storage, data transfer and sharing, protection of human subjects, ownership, trademark, etc; IRB expertise in the oversight of the protection of human subjects; commercialization and licensing experts to assist with trademark, limited liability company formation, and business agreements; business partners or vendors who will assist in the financial and infrastructure support to scale and manage the mHealth app; and finally, individuals with lived experience to inform app development and ongoing tailoring.

#### Exploratory Preliminary Pilot Studies

Preliminary studies exploratory in nature to inform the gaps, needs, facilitators and barriers, functionality, design, feasibility, usability, and acceptability among the targeted population are essential. This could require a substantial amount of time, intramural and extramural funding, and institutional support. We suggest that all preliminary studies except for literature reviews should involve individuals with lived experience using a combination of qualitative and quantitative methods. We also recommend using our methods to first conduct a search of what apps are already targeted at the population of interest to better understand gaps. Then, piloting apps that are already on the market, with permission from the owner, would be the next step. Testing existing apps that have functionality or focus on a topic relevant to your population would be an ideal way to conduct preliminary feasibility, usability, and acceptability testing prior to actual app development.

#### Geographic Location

Geographic location is another factor to consider. Our population was in Texas and on a form of community-based criminal legal oversight outside the confinements of jail or prison, making participant interaction and intervention testing more feasible. Local, state, and federal laws and institutional or environmental barriers that may hinder feasibility, usability, and acceptability within a target population must be considered. In addition, community partnerships with recruitment sites are essential for accessing the targeted population.

#### Methodological Aspects That May Be Transferable or Context-Specific

There are some methodological aspects that could be transferable to other populations with similar overlapping qualities such as criminal legal involvement, the social determinants of health, women’s health issues pertaining to violence, perinatal substance use disorder, parenting, pregnancy, childbirth, and postpartum. For instance, pregnancy-related deaths can arise from factors at the individual level (eg, maternal age, substance use, mental health conditions, and chronic diseases), the community level (eg, unstable housing, inadequate access to health care, lack of reliable transportation, and living in rural areas), and the institutional level (eg, limited policies or procedures or biased care) [67], especially among women with criminal legal system involvement.

Our preliminary studies [38-40,45] and current data being collected in pilot tests [Ihle, unpublished data, 2026] reflect data from other authors, which provide insight from women on community supervision having health compilations and adverse experiences or outcomes that could have been mitigated had they had access to timely and cost-effective health care [26,29,68,69]. Therefore, we can assume that mHealth apps with AI chatbot technology that uses a systematic method guided by the ISR framework to direct design, implementation, and evaluation show promise in effectively heightening self-efficacy among groups with similar or overlapping risks to their overall health.

#### Cost Estimates for Development and Maintenance

The costs may vary depending on the type of design, functionality, preliminary testing needed, and partnership agreements. However, preliminary studies within our methodological approach ranged from in-kind effort (US $0) upward to US $350,000 to support salaries, participant incentives, and data collection and analysis. In addition, vendor fees for tailoring the images (US $100-$1000) and covering the costs for developing, managing, and scaling (up to US $50,000) of the intervention can be estimates others can use to gauge their work, time, and budgets on.

#### Scalability Challenges

Finally, scalability challenges will arise, especially if development and testing are within academic settings. For instance, within academic settings, funding to cover the development, management, and scaling of the intervention will most likely come from intramural or extramural grant funding with instances that may cause delays in between funding. In addition, limited salary support and protected time of investigators combined with the overturn of staff comprised of students who may graduate may impede the development and testing efforts of an mHealth intervention. Therefore, it is essential to get guidance and support from institutional commercialization departments to find vendor or business partnerships to help in financial and development support to manage, sustain, and scale the technology beyond the parameters of an academic institution.

### Comparison With Prior Work: Ethical Considerations

#### Overview

Considering JUN is a health-related app that gives guidance around sensitive issues such as mental health, violence, criminal legal system involvement, and perinatal health, the ethical standards must be applied during app development and management specific to the health care field and protections of human subjects. Overall, the ethical standards must encompass the basic principles of beneficence and nonmaleficence. To maintain these principles, JUN operates in the following manner around crisis detection, privacy protection, and fallback mechanisms.

#### Crisis Detection

JUN and its incorporated AI chatbot are clearly stated to not substitute for in-person medical care, diagnose, nor counsel. When the JUN AI chatbot managed by Scalable Care detects indications of critical health or safety-related symptoms that may harm an individual or others, research staff (in this case, the principal investigator [ADI]) is automatically notified, and the user is directed to appropriate emergency services. The team trained its crisis detection system with harm to self, harm to others, or suicide as priority categories, overriding other possible detection categories (eg, emotional valence or life situation). Tolerance limits were chosen with preference for type II error over type I error, as a false positive (eg, detecting a crisis where there is none) is largely harmless, while a false negative may leave a person in crisis without support [70]. Further, support for this priority comes from work, showing that candidly asking about suicidal ideation decreases risk [71]. The JUN AI chatbot, in our case study, can continue conversations following a user’s disclosure of ideation followed by denial of an immediate crisis. Should a user describe an immediate crisis, the AI chatbot will pause the conversation and direct the user to appropriate channels of health care or emergency support.

#### Privacy Protection

Private health data are stored securely, and access is strictly limited as outlined and agreed upon between the university-vendor Business Associate Agreement and IRB approved protocol. The “Journal” feature, which provides functions around symptom tracking, statistics, trends, and AI-led feedback, potentially contains personal health data submitted by users in JUN. Due to the vulnerable nature of the target population, cases of phone theft, browser snooping, and similar violations of privacy by individuals gaining unwanted access to the user’s device are probable. To mitigate this risk, access to the “Journal” feature requires a secondary login step even for registered users.

#### Fallback Mechanisms

Educational information on JUN is factual, verified, and represents the current state of knowledge of the respective fields and is presented in a way that is approachable and appropriate for the target population. Operational efficiency may not be prioritized when considering ethics; however, the public has displayed trends and voiced serious ethical concerns [72] regarding the widespread, insidious, or overabundant use of AI, as well as associated data centers’ use of electricity and water [73], environmental damage [74], and public health risks. While there are efforts to expand AI tools in a more sustainable fashion, the current regulatory regime is a patchwork of laws and proposals at the national, state, and local levels [71]. Each of these issues requires further interrogation and is beyond the scope of this study.

However, no discussion of the ethical burden borne by mental health service providers’ use of AI tools could stand scrutiny without acknowledging the rapidly growing data center elephant in the room. To address this, the JUN chatbot uses a multiagent structure, which limits its large model use to tasks that cannot be accomplished by its smaller, purpose-trained models. These smaller models are used to detect the emotional valence of the user’s input, life situation, or presenting concern including a crisis or state of emergency—recommending relevant education or health, safety, or social-related resources.

### Limitations

Despite the novelty of our methodological approach used in this paper, limitations exist that we must consider. First, preliminary and current pilot testing that has informed the design and tailoring of JUN has been conducted within one geographical region of South-Central Texas, which is predominantly Hispanic, potentially limiting generalizability and applicability among other populations. Most of our data are cross-sectional in nature, except for a microlongitudinal study across 3 months. Another limitation is the small training dataset of 220 sentences; however, using techniques such as fine-tuning [75] and in-context learning [76], only a minimal number of examples are necessary to adjust these models to perform domain-specific tasks with higher accuracy than hand-tailored prompts.

Another limitation is the sustainability and long-term maintenance concerns after research funding ends, which is why business agreements like our methods with vendor partnerships to help with managing and scaling the technology and to inform commercialization are essential. Potential for harm is also another limitation to consider if the AI chatbot provides inappropriate advice in crisis situations. We addressed this by having members of our team consistently test the AI chatbot in our beta-testing site using language and phrases that suggest self-harm or harm to others. We also set the algorithm to be more sensitive around type II error over type I error, such as being overly sensitive to deter a crisis where there is none, to be the best route to ensure that support is given to participants when needed. Another limitation is the consideration of how rapidly LLM technology is evolving; therefore, our methodological approach may not be the most efficient method of developing and testing an AI chatbot in future projects. Therefore, the continuation of developing and informing methodological approaches is essential.

We also did not have our target population involved in creating the sentences for training generation; however, we used preliminary data to inform the sentences and topics for our categories and challenges within our methods to train our AI chatbot. Finally, there are potential biases from our university-vendor collaboration and conflicts of interest, considering that the vendor who has helped develop, tailor, manage, and scale our technology is a for-profit company. We have mitigated biases by making our methods informed by preliminary studies, which engaged women with lived experience. Additionally, our methodological approach is supported by recent literature by authors without biases or conflicts of interest. Despite these limitations, our methodological approach addresses some of the gaps of current literature and may be useful to help make assumptions toward other populations and case studies. Larger and more controlled clinical trials across longer durations of time must be implemented to fully understand the effectiveness of AI chatbots among underserved populations.

### Conclusions

Health disparities such as maternal morbidity and mortality among childbearing women remain high in the United States, especially among those with risks associated with the criminal legal system. This research addresses how iteratively applying the 3 phases of the ISR framework in the design, implementation, and testing of an mHealth app may enhance the AI chatbots’ algorithm to provide tailored feedback to appropriately address the complex needs of underserved populations such as the women involved with the criminal legal system used in our case study.

AI chatbot technology has the potential to revolutionize health care access and strengthen the ability to customize and deliver adaptive health care to vulnerable populations. However, the complex and intersecting needs of underserved populations require focus to address gaps, including design data, relevant user content, data security, and privacy. Underserved groups experience compounded social and health inequities aggravated by social determinants such as low socioeconomic status, undiagnosed or chronic disease, discrimination, and low digital literacy [6]. Genuinely addressing the needs of the target population requires deliberate cocreation input that includes the people at the margins of health care [5].

mHealth app content must extend beyond generic scopes of practice, becoming contextualized and specific to the unique and complex needs of underserved groups [1]. Therefore, researchers and computer engineering teams during the design, tailoring, and testing process must collaborate with underserved communities to gain insight regarding the appropriate cultural terminology and nuances specific to local values and norms [5]. As such, mHealth apps’ relevance and design must include how the content is delivered, the accessibility of content, combined with how users engage with the intervention’s interface [8].

Another factor researchers and engineers must consider during mHealth app development is the privacy and security concerns that may deter trust, acceptability, and usability among underserved groups from interacting with digital technology [7]. Similarly, scholars must consider technological tethering based on opaque data systems, whereby AI tools may monitor data to penalize underrepresented groups [77]. Implementing ethical safeguards in legal-binding Scope of Work, Business Associate Agreements, and IRB protocols, including language-friendly consent and transparent privacy policies, may educate and gain the trust of users [77]. Altogether, inclusive data and mHealth app design promote empowerment and trust. The ethical design of mHealth apps must intentionally consider intersectionality; otherwise, AI chatbot technology may have limited efficacy to address the needs of underserved groups.

## Appendix

Multimedia Appendix 1 Study 1: The Experiences of Latina Women with Histories of Incarceration, Tables S1 Participant Interview Guide; S2 Maternal Demographics.

Multimedia Appendix 2 Study 2: Review of mHealth Applications Specific to the health disparities of women on community supervision; Table S1: Study Design, Sample, Objectives, & Conclusions; Table S2: Measures, Intervention, Analytic Approach.

Multimedia Appendix 3 Study 3: Intervention Development Study to Test the Feasibility, Usability, and Acceptability of mHealth Apps; Table S1: Features of the mHealth apps; Table S2: Interview guide using Social Cognitive Theory as the guiding framework; Table S3: Length of abuse/violence and reasons for not reporting; Table S4: Principles of SCT and Exemplars.

Multimedia Appendix 4 Rigor Cycle Study 1: Exploring JUNTM Amongst Women with Histories of Community Supervision; Table S1: Rigor Cycle Study 2 Exploring JUNTM Social Cognitive Theory Interview Guide.

Multimedia Appendix 5 Rigor Cycle Study 2: Testing JUN Amongst Pregnant Women; Table S1: Health Belief Model Interview Amongst Pregnant Women with and without Community Supervision.

Multimedia Appendix 6 Rigor Cycle Study 3: Reliability and Validity Testing of the JUNTM AI chat-bot; Table S1: Interview guide for the pre and post-intervention AI chaat-bot training sessions.

## Abbreviations

AI: artificial intelligence
IRB: institutional review board
ISR: Information Systems Research
LLM: large language model
mHealth: mobile health
PRISMA: Preferred Reporting Items for Systematic Reviews and Meta-Analyses

## Data Availability

The datasets used and/or analyzed during this study are available from the corresponding author on reasonable request.

## References

[1] Anderson-Lewis C, Darville G, Mercado RE, Howell S, Di Maggio S (2018). mHealth technology use and implications in historically underserved and minority populations in the United States: systematic literature review. JMIR Mhealth Uhealth.

[2] (2025). Mobile fact sheet. Pew Research Center.

[3] Babu M, Lautman Z, Lin X, Sobota MHB, Snyder MP (2024). Wearable devices: implications for precision medicine and the future of health care. Annu Rev Med.

[4] Wells C, Spry C (2022). An overview of smartphone apps. Can J Health Technol.

[5] Armaou M, Araviaki E, Musikanski L (2019). eHealth and mHealth interventions for ethnic minority and historically underserved populations in developed countries: an umbrella review. Int J Community Well-Being.

[6] Geana MV, Anderson S, Lipnicky A, Wickliffe JL, Ramaswamy M (2021). Managing technology, content, and user experience: an mHealth intervention to improve women's health literacy after incarceration. J Health Care Poor Underserved.

[7] Marra AR, Nori P, Langford BJ, Kobayashi T, Bearman G (2023). Brave new world: leveraging artificial intelligence for advancing healthcare epidemiology, infection prevention, and antimicrobial stewardship. Infect Control Hosp Epidemiol.

[8] Carreiro S, Newcomb M, Leach R, Ostrowski S, Boudreaux ED, Amante D (2020). Current reporting of usability and impact of mHealth interventions for substance use disorder: a systematic review. Drug Alcohol Depend.

[9] Patil MV, Shree PS, Singh P (2021). AI based healthcare chat bot system. Int J Sci Eng Res.

[10] Collins LM (2018). Introduction to adaptive interventions. Optimization of Behavioral, Biobehavioral, and Biomedical Interventions.

[11] Yang T, Zheng HX, Cao S, Jing M, Hu J, Zuo Y, Chen Q, Zhang J (2025). Harnessing an artificial intelligence-based large language model with personal health record capability for personalized information support in postsurgery myocardial infarction: descriptive qualitative study. J Med Internet Res.

[12] Gupta A, Hathwar D, Vijayakumar A (2020). Introduction to AI chatbots. Int J Eng Res Technol.

[13] Mei X, Li J, Li ZS, Huang S, Li L, Huang Y, Liu J (2022). Psychometric evaluation of an adverse childhood experiences (ACEs) measurement tool: an equitable assessment or reinforcing biases?. Health Justice.

[14] Mei Q, Xie Y, Yuan W, Jackson MO (2024). A Turing test of whether AI chatbots are behaviorally similar to humans. Proc Natl Acad Sci USA.

[15] Cronholm S, Gobel H (2016). Evaluation of the information systems research framework: empirical evidence from a design science research project. Electron J Inf Syst Eval.

[16] Hevner AR (2007). A three cycle view of design science research. Scand J Inform Syst.

[17] Fink DA, Kilday D, Cao Z, Larson K, Smith A, Lipkin C, Perigard R, Marshall R, Deirmenjian T, Finke A, Tatum D, Rosenthal N (2023). Trends in maternal mortality and severe maternal morbidity during delivery-related hospitalizations in the United States, 2008 to 2021. JAMA Netw Open.

[18] Saluja B, Bryant Z (2021). How implicit bias contributes to racial disparities in maternal morbidity and mortality in the United States. J Womens Health (Larchmt).

[19] Wang E, Glazer KB, Howell EA, Janevic TM (2020). Social determinants of pregnancy-related mortality and morbidity in the United States: a systematic review. Obstet Gynecol.

[20] Wang S, Rexrode KM, Florio AA, Rich-Edwards JW, Chavarro JE (2023). Maternal mortality in the United States: trends and opportunities for prevention. Annu Rev Med.

[21] Sufrin C, Beal L, Clarke J, Jones R, Mosher WD (2019). Pregnancy outcomes in US prisons, 2016–2017. Am J Public Health.

[22] Sufrin C, Jones RK, Mosher WD, Beal L (2020). Pregnancy prevalence and outcomes in U.S. jails. Obstet Gynecol.

[23] Heimer K, Malone SE, De Coster S (2023). Trends in women's incarceration rates in US prisons and jails: a tale of inequalities. Annu Rev Criminol.

[24] Kajstura A, Sawyer W (2024). Women's Mass Incarceration: The Whole Pie.

[25] Diaz CL, Rising S, Grommon E, Northcutt Bohmert M, Lowder EM (2022). A rapid review of literature on factors associated with adult probation revocations. Corrections.

[26] Hawks L, Wang EA, Howell B, Woolhandler S, Himmelstein DU, Bor D, McCormick D (2020). Health status and health care utilization of US adults under probation: 2015–2018. Am J Public Health.

[27] Lorvick J, Comfort M, Kral AH, Lambdin BH (2018). Exploring lifetime accumulation of criminal justice involvement and associated health and social outcomes in a community-based sample of women who use drugs. J Urban Health.

[28] Lorvick J, Comfort ML, Krebs CP, Kral AH (2015). Health service use and social vulnerability in a community-based sample of women on probation and parole, 2011–2013. Health Justice.

[29] Lorvick J, Hemberg JL, Browne EN, Comfort ML (2022). Routine and preventive health care use in the community among women sentenced to probation. Health Justice.

[30] Lynch SM, Kaplan S (2025). Examining trauma-related shame and trauma coping self-efficacy as predictors of PTSD in women in jail. Soc Sci.

[31] Crawford AD, Geramifar LL, McGlothen-Bell K, Salisbury E (2025). A reproductive justice investigation of utilizing digital interventions among underserved populations with criminal legal system supervision: policy brief. Nurs Outlook.

[32] Crawford AD, Linder GL, McGlothen-Bell K, Salisbury E (2025). Reproductive justice investigation of digital intervention use amongst women with criminal justice system oversight: policy brief. Target J Nurs Outlook.

[33] Lawrence TI (2022). The effects of adverse childhood experiences (ACEs), mental illness, and personality differences on attitudes toward self-efficacy among females on parole/probation. Int J Offender Ther Comp Criminol.

[34] Liu L, Lazazzara G, Meldrum RC (2023). The nexus of violent victimization, mental health, and employment: findings from a sample of post-incarcerated individuals. J Interpers Violence.

[35] Crawford AD, Zettler H, Braddy S, Hayward M, Howe R, Rowe J, McGrath JM (2025). Community supervision engagement to examine health outcomes: a scoping review. Public Health Nurs.

[36] Van Deinse TB, Zielinski MJ, Holliday SB, Rudd BN, Crable EL (2023). The application of implementation science methods in correctional health intervention research: a systematic review. Implement Sci Commun.

[37] Crawford AD, Testa A, Darilek U, Howe R, McGrath JM, Shlafer R (2024). Perinatal health outcomes among women on community supervision: a scoping review. J Correct Health Care.

[38] Crawford AD, Hutson TS, Kim M (2023). Mobile health applications addressing health disparities for women on community supervision: a scoping review. Subst Use Misuse.

[39] Crawford AD, McGlothen-Bell K, Marsh LN, Cleveland LM (2022). "We're Still Human": a reproductive justice analysis of the experiences of criminalized Latina mothers. J Aggress Maltreatment Trauma.

[40] Crawford AD, Salisbury EJ, McGrath JM (2024). An intervention development study of an mHealth app to manage women's health and safety while on probation. Health Justice.

[41] Sandelowski M (2000). Whatever happened to qualitative description?. Res Nurs Health.

[42] Sandelowski M (2010). What's in a name? Qualitative description revisited. Res Nurs Health.

[43] Ross L (2020). Understanding reproductive justice. Feminist Theory Reader.

[44] Crawford AD (2021). The experiences of Latina mothers impacted by incarceration [doctoral dissertation]. The University of Texas Health Science Center at San Antonio.

[45] Crawford AD, McGlothen-Bell K, Cleveland LM (2022). "I did whatever they wanted me to do": a qualitative secondary analysis using reproductive justice to explore sexual violence among justice-involved Latina mothers. BMC Public Health.

[46] Crawford AD, Ricks TN, Bell KM, McGrath JM, Abbyad C, Polinard E, Cleveland LM (2024). Conditions that influence coping mechanisms in Latina mothers affected by incarceration: a secondary analysis using the vulnerability framework. Res Nurs Health.

[47] Page MJ, McKenzie JE, Bossuyt PM, Boutron I, Hoffmann TC, Mulrow CD, Shamseer L, Tetzlaff JM, Akl EA, Brennan SE, Chou R, Glanville J, Grimshaw JM, Hróbjartsson A, Lalu MM, Li T, Loder EW, Mayo-Wilson E, McDonald S, McGuinness LA, Stewart LA, Thomas J, Tricco AC, Welch VA, Whiting P, Moher D (2021). The PRISMA 2020 statement: an updated guideline for reporting systematic reviews. BMJ.

[48] Julian S (2023). Nice Intimacy Tracker.

[49] Potter SJ, Moschella EA, Smith D, Draper N (2020). Exploring the usage of a violence prevention and response app among community college students. Health Educ Behav.

[50] Potter SJ, Moschella EA, Demers JM, Lynch M (2021). Using mobile technology to enhance college sexual violence response, prevention, and risk reduction efforts. J Technol Human Serv.

[51] Lewis P, Perez E, Piktus A (2021). Retrieval-augmented generation for knowledge-intensive NLP tasks. ArXiv. Preprint posted online on April 12, 2021.

[52] Choudhury MD, Pendse SR, Kumar N (2023). Benefits and harms of large language models in digital mental health. ArXiv. Preprint posted online on November 7, 2023.

[53] Bender EM, Gebru T, McMillan-Major A, Shmitchell S (2021). On the dangers of stochastic parrots: can language models be too big.

[54] Fitzpatrick KK, Darcy A, Vierhile M (2017). Delivering cognitive behavior therapy to young adults with symptoms of depression and anxiety using a fully automated conversational agent (Woebot): a randomized controlled trial. JMIR Ment Health.

[55] Darcy A, Beaudette A, Chiauzzi E, Daniels J, Goodwin K, Mariano TY, Wicks P, Robinson A (2023). Anatomy of a Woebot® (WB001): agent guided CBT for women with postpartum depression. Expert Rev Med Devices.

[56] Guo Z, Lai A, Thygesen JH, Farrington J, Keen T, Li K (2024). Large language models for mental health applications: systematic review. JMIR Ment Health.

[57] Moore J, Grabb D, Agnew W (2025). Expressing stigma and inappropriate responses prevents LLMs from safely replacing mental health providers.

[58] Grové C (2020). Co-developing a mental health and wellbeing chatbot with and for young people. Front Psychiatry.

[59] Sudhi V, Bhat SR, Rudat M, Teucher R (2024). RAG-Ex: a generic framework for explaining retrieval augmented generation.

[60] Yang X, Cheng W, Zhao X, Yu W, Petzold L, Chen H (2023). Dynamic prompting: a unified framework for prompt tuning. ArXiv. Preprint posted online on May 27, 2023.

[61] Hesham A, Hamdy A (2024). Fine-tuning GPT-4o-mini for programming questions generation.

[62] Crawford AD, Slavin R, Tabar M, Radhakrishnan K, Wang M, Estrada A, McGrath JM (2024). Methodological approaches in developing and implementing digital health interventions amongst underserved women. Public Health Nurs.

[63] Rosenstock IM, Strecher VJ, Becker MH (1994). The health belief model and HIV risk behavior change. Preventing AIDS: Theories and Methods of Behavioral Interventions.

[64] Bandura A (1986). Social Foundations of Thought and Action: A Social Cognitive Theory.

[65] Bandura A (1999). Social cognitive theory of personality. Handbook of Personality.

[66] Federici S, Borsci S (2010). Usability Evaluation: Models, Methods, and Applications.

[67] Ahn R, Gonzalez GP, Anderson B, Vladutiu CJ, Fowler ER, Manning L (2020). Initiatives to reduce maternal mortality and severe maternal morbidity in the United States. Ann Intern Med.

[68] Karlsson ME, Zielinski MJ (2020). Sexual victimization and mental illness prevalence rates among incarcerated women: a literature review. Trauma Violence Abuse.

[69] Belisle LA, Salisbury EJ, Cowell Mercier M (2023). Gender-responsive probation during the COVID-19 pandemic: learning from justice-involved women and their supervising officers. Victims Offenders.

[70] Guidi G, Dominici F, Gilmour J, Butler K, Bell E (2024). Environmental burden of United States data centers in the artificial intelligence era. ArXiv. Preprint posted online on November 14, 2024.

[71] (2025). AI Watch: global regulatory tracker—United States. White & Case, LLP.

[72] Dazzi T, Gribble R, Wessely S, Fear NT (2014). Does asking about suicide and related behaviours induce suicidal ideation? What is the evidence?. Psychol Med.

[73] Kerr D (2024). How Memphis Became a Battleground over Elon Musk's xAI Supercomputer.

[74] Kerr D (2025). Elon Musk's xAI Accused of Pollution over Memphis Supercomputer.

[75] Bakker M, Chadwick M, Sheahan H (2022). Fine-tuning language models to find agreement among humans with diverse preferences. https://proceedings.neurips.cc/paper_files/paper/2022/file/f978c8f3b5f399cae464e85f72e28503-Paper-Conference.pdf.

[76] Wang X, Zhu W, Saxon M, Steyvers M, Wang MY (2023). Large language models are implicitly topic models: explaining and finding good demonstrations for in-context learning. https://openreview.net/pdf?id=HCkI1b6ksc.

[77] Gipson Rankin SM (2021). Technological tethereds: potential impact of untrustworthy artificial intelligence in criminal justice risk assessment instruments. Wash Lee L Rev.

