# Supplementary material for: Training an AI Chatbot to Manage Health in Underserved Populations: Methodological Approach
Source: JMIR AI. 2026 Apr 1;5:e84145. doi: 10.2196/84145 (PMC13085989; doi:10.2196/84145)
Supplement: Multimedia Appendix 1 [file ai_v5i1e84145_app1.pdf]

Appendix 1

Study 1: The Experiences of Latina Women with Histories of Incarceration

Table S1. *Participant Interview Guide*

	**Questions**
**1**	Describe your experiences of being a mother following arrest?
**2**	What are the biggest challenges of parenting after arrest?
**3**	Describe a typical day in your life?
**4**	What are your barriers to healthcare?
**5**	What would make it easier for moms following arrest?
**6**	What is most important to you?
**7**	What worries you in regard to your children
**8**	What worries do you have regarding your health or the health of your family?
**9**	What about your health is most important to you?
**10**	Can you share with me what you want most for the future?
**11**	Is there something else you think I should know that I haven't already asked?
**12**	Probe questions<br>a) Some women have described instances where they were not in control of their bodies when seeking healthcare, can you describe any experience where you did no feel in control of your body?

b) Women have described being forced to engage in sexual acts against their will or were touched inappropriately following arrest, can you describe any time this has happened to you as well?

Table S2. *Maternal Demographics*

**Participants (*N* = 12)**	***n***	**Range**	**Mean**
Age			
<31	1		
32–38	5		
Older than 39	6		
Ethnicity			
Relationship Status			
Single	7		
Living with partner	4		
Married	1		
Number of Children			
≤4	6	1–4	2.5
>5	6	5–8	5.5
Highest Level of Education Co pleted			
Middle school	6		
High school	1		
Associates/ Certification	4		
Bachelors	1		
Employment Status			
Unemployed	10		
Part-time	0		
Full-time	2		
Annual Income			

Less than $9,000	9		
$10,000–$19,000	2		
$20,000–$29,000	0		
$30,000–$39,000	1		
Number of Times Arrested		1–19	9
Nature of Arrests			
Low-level drug	8		
Petty theft	7		
DUI	1		
Traffic tickets	2		
Prostitution	2		
Assault	1		
County jail	12	18 hr to 22 months	11 months
State prison	2	1 month to 2.2 years	13 months
Federal prison	0		
Community supervision	11	2–5 years	2.5 years
Transition home	2	9–12 months	10.5 months
Misdemeanor	11		
Felony	12		
