# Supplementary material for: Training an AI Chatbot to Manage Health in Underserved Populations: Methodological Approach
Source: JMIR AI. 2026 Apr 1;5:e84145. doi: 10.2196/84145 (PMC13085989; doi:10.2196/84145)
Supplement: Multimedia Appendix 2 [file ai_v5i1e84145_app2.docx]

**Appendix 2**

**Study 2: Review of mHealth Applications Specific to the health disparities of women on community supervision**

**Table S1.** *Study Design, Sample, Objectives, & Conclusions (N=*5*)*

**# Author, Year**

**Design & Sample**

**Objectives Conclusions**

1. Johnson et

al., 2016

1. Johnston et

al., 2019

Pilot test

*N*=30

Men & women with alcohol use disorder in drug court

Quasi-Experimental *N*=198

Appalachian women

Test the accessibility and usability of an app called A-CHESS for recovery support and relapse prevention.

To evaluate the retention in a MAT program using the A-CHESS app in underserved populations.

*StaySafe* is convenient, alleviates burden, and efficient by being able to be self-administered, and implemented by staff with minimal training or effort. The role-play videos, interactive features and games increases usability and engagement. Repetition of the WORKIT app is integrated in the Staysafe. The usability of the app on a tablet heightens confidentiality. A smartphone version is recommended for future studies to increase accessibility and usability of the app. Other content areas such as women’s reproductive health issues can be integrated within he app.

A-CHESS is effective at decreasing the number of risky drinking days for people who have been recently discharged from residential treatment for AUD. It is being piloted in other populations, including pregnant and postpartum women, veterans, people who are deaf or hard of hearing, and people identified in primary care settings as being in need of SUD treatment. Virtual discussion groups is imperative in keeping participants active, preventing dropout, and promoting positive communication skills. Drug court participants showed acceptability and usability of the app during recovery. Drug court participant’s use of the app differs than those outside of drug court.

The effectiveness of the tool in reducing substance us severity is suggested in future research.

1. Lehman et.

al, 2021

1. Muroff et al., 2017
2. Shrestha et

al., 2017

Quantitative Observational Cohort *N*=511

men & women on community supervision and residential treatment settings

Cross sectional *N*=79

Latino(a) men & women

Cross sectional *N*=400

Men & women

Evaluating the efficacy of an app called StaySafe, to assist with STI prevention and sexual health promotion while on probation.

Evaluate the usability, cultural competence and rates of relapse of ppl using CASA-CHESS, a modified version of A-CHESS app designed for Latino(a)s with SUD on an MAT program.

Evaluate the interested and usability of people on an MAT program in the use of an app for sexual health behavior change, STI prevention and MAT reminders/management.

A-CHESS, used with intensive outpatient treatment, has the ability to improve long-term recovery rates for women with an SUD in underserved or rural/isolated regions. Retention improved from: 1) social and communication features to connect participants with their counselors; 2) the Weekly Survey regularly informing the counselor of the participant’s status (negative and positive variables) helped prompt treatment; 3) the use of Discussion Groups and Private Messages which gave a sense of community. Future studies should investigate the key tenets of self-determination theory: (1) feeling competent, (2) feeling connected to others, and (3) feeling internally motivated rather than coerced. A-CHESS helps with connectedness. Women, more than men, use social media and technology, even when they are not confident with using the tool.

Preliminary results displayed that Latinos will use a smartphone app after residential treatment. Competence-related services decreased, possibly because they are not as dynamic as relatedness or autonomy services. Low functional literacy may have affected use of some CASA- CHESS features and usability. There is a limited amount of mHealth apps that are culturally and linguistically tailored for Spanish-speaking Latinos with alcohol and other drug disorders/mental disorders. Policy implications include ensuring that mHealth technology is affordable and accessible. mHealth aps allows patients to inform providers of return to use, while offering clinicians time sensitive behavioral health information.

Those with cognitive impairment and taking medications showed a higher interest in mHealth services to receive medication reminders. This suggests this population is concerned about the risk of HIV infection but also motivated to participate in their health and treatment plan. Future research needs to explore how at-risk PWUD perceive and respond to their HIV risks. Also, important consideration related to security, privacy, and ease of use must be investigated. Preferences for design and functionality of mHealth-based HIV prevention are needed

as well as development, implementation, and evaluation of intervention strategies.

**Table S2:** *Measures, Intervention, Analytic Approach*

**# Author, Year Measures or Intervention Analytic Approach**

1. Lehman et al., 2021

**StaySafe:** twelve 10 min self-administered sessions on a tablet. Gives an overview of how to navigate through the *StaySafe* app, followed by a demonstration of a WORKIT exercise

**HIV Knowledge Confidence, Avoiding Risky Sex, HIV Services & Testing, and Risk Reduction Skills.** Each of the scales, except for *HIV Services & Testing*, included items that assessed how knowledgeable (K) the participant felt about the topic, how confident (C) they were about their knowledge, and how motivated (M) they were to act on the knowledge. *HIV Services & Testing* included only knowledge and motivation items. The study included knowledge, confidence, and motivation components for each scale in an overall score and examined them as separate subscales.

**HIV Knowledge Confidence** scale has 13 items (alpha = 0.93), and sample items include “You know enough to teach others what they should do if they think they have been exposed to HIV” (K), “You feel very confident that you could be a role model for others in helping reduce HIV risks” (C), and “You are totally committed to helping your friends and/or family avoid HIV/AIDS” (M). Coefficient alpha reliabilities for the three subscales ranged from 0.75 to 0.88.

**Avoiding Risky Sex scale** has 13 items (alpha = 0.93 and

There were 3 sets of analyses. 1) report characteristics of the sample and *StaySafe* participation, in terms of the number of sessions completed. 2) compared the *StaySafe* and SP groups on baseline and postintervention measures using SAS Proc GLM separately for the community and residential samples: (a) demographic and background variables, (b) baseline measures of the outcome variables to check for group equivalence using chi-square statistics for dichotomous and categorical variables and *t*-tests for continuous variables. 3) compared the *StaySafe* and SP groups on outcome variables from the postintervention surveys using the appropriate baseline measure as a covariate.

ranged from 0.81 to 0.84 for the three subscales) and includes items such as “During the past month, you have learned about what situations might lead you to make a poor decision about risky sex” (K), “During the past month, your confidence in managing emotions in sexual situations in the real world has increased” (C), and “During the past month, you have become more motivated to protect your sexual partner from HIV risk in the real world” (M).

**HIV Services & Testing scale** consists of seven items (alpha

= 0.81 for the full scale; 0.62 to 0.79 for the knowledge and motivation subscales, respectively). Sample items include “During the past month, you have become more knowledgeable about how to get HIV services in the real world” (K) and “You will get tested for HIV if you think that you might have been exposed” (M). **Risk Reduction Skills scale** comprises 14 items (alpha = 0.91, ranging from 0.65 to 0.82 for the three subscales) and includes items such as “During the past month, you have a better understanding of how your *should* and *wants* can conflict in the real world” (K), “During the past month, you have become more confident in balancing your *should* and *wants* in the real world” (C), and “During the past month, your motivation to avoid personal HIV risks in the real world has increased” (M). **Confidence & Motivation scales** all items use a 5-point Likert-type response scale ranging from 1 = Disagree Strongly to 5 = Agree Strongly. We reflected items worded in the opposite direction from the scale construct by subtracting the score from 6. Study staff then computed scale scores by calculating the average score for items within the scale then multiplying the average score by 10 to obtain a range from 10 to 50. Scores above 30 indicated at least some agreement with the scale construct and scores below 30 indicated at least some disagreement.

Decision Making scales:

**Rational DM scale** consists of five items (alpha = 0.84), including “I make decisions in a logical and systematic way” and “I explore all of my options before making a decision.” This scale is relevant for the “O” in the WORKIT schema and involves exploring options for a problem. **Dependent DM scale** has five items (alpha = 0.79) with statements such as, “I often need the assistance of other people when making important decisions” and “I rarely make important decisions without consulting other people.” This scale is relevant for the “W” component of WORKIT (who will be affected and who can help with a problem).

**TCU DM** scale has nine items (alpha = 0.79). Representative items include “You plan ahead,” You think about what causes your current problems,” and “You think of several different ways to solve a problem.” This scale represents a more general view of decision-making.

Talk outcomes: The study team created talk variables from a series of 12 questions created for this study that asked about whether the individual talked to their probation officer, counselor, friend/trusted advisor, or family member about better decision-making, HIV risks, and HIV prevention/ treatment. The dependent variables were dichotomies of whether talk occurred with that person(s) or addressed that subject matter.

Getting tested: We asked how many times the participant had been tested for HIV, a sexually transmitted infection, or hepatitis B or C in the past year (at baseline) and at postintervention.

**WORKIT:** Nine sessions - start with choosing from a list of 11 problem themes related to (1) people (e. g., “Asking a partner about his or her HIV testing”); (2) places (e.g., “Favorite high-risk places to hang out”); or (3) things (e.g., “Practicing safe sex”). Then, vignettes with actors are shown

1. Johnson et al., 2016

who work through the scenario. Participants are asked to select from a list of choices on how to trouble shoot the issue, there is also “pop ups” with health fact, lastly there is a maze game that progresses when each answer is answered correctly.

**Participant Choice (PC):** Three sessions- several information-based activities around HIV and health risks; uses three different activities: (1) a Center for Disease Control video giving information about HIV/AIDS, (2) HIV/AIDS facts coupled with a video discussion about HIV medication therapy or someone affected by HIV/AIDS talking about their experiences, and (3) the maze question game with additional HIV- related content. Participants chose one or more of the activities. Each of the Participant Choice sessions followed two WORKIT sessions in the weekly sequence, which provided for variation between tablet sessions.

A web server gathered data on each participant’s use the A-CHESS app. The date and time the participant logged in, the A-CHESS ser-vices selected, length of time the service was open, the number of pages viewed, and whether the participant sent or received messages were tracked.

A-CHESS tools: information services (recovery information, a self-help meetings list, news about substance use and recovery, activities such as games, guided relaxation recordings and podcasts about recovery, interactive communication, such as messaging and discussion boards, and assessment and feedback tools). Participants received two instant messages per day: a “thought for the day,” to be motivational, elicit us e of app or refer to professional support if well-being was threatened.

Each tool within the app is designed to enhance motivation, provide skills or remind participants of skills they have learned in treatment, or use skills to increase competence, and quickly access social support and further develop RE virtually based

Use of the app was analyzed by different types of services. Analysis of who communicated with whom and how often; communication between participants in the discussion board were used to create a numerical description of the network from identifying the sender, responders, and number of posts. The social network analysis conducted used NodeXL (V1.0.1.245) to calculate traditional social network measures, such as centrality (three types: closeness, betweenness, and eigenvector) and density of networks. These measures were used to draw diagrams of the network that highlight who is most central, frequency of communications and how dense.

Graphs of two networks were drawn by using the force-directed Harel-Koren fast multiscale layout. To distinguish the influence of program staff and

1. Johnston et al., 2019

on the constructs of Social Determination Theory (SDT). The version of the app in this study was modified by adding more information on drug abuse in the recovery information and newsfeed sections.

A-CHESS app with services: (1) communication with professionals and peer support groups (2) timely monitoring, feedback- related assessment, and links to interventions addressing relapse risk; (3) reminders and alerts to encourage adherence to therapeutic goals; (4) one-touch communication with the patient’s counselor and/or case manager; (5) addiction-related educational materials and tools; and (6) customizable location-based resources (eg, alerts that would display if a patient neared a high-risk location such as a bar or supplier she used to frequent). Screen shots of A-CHESS are available at [http://chess.wisc.edu/achess-archive/.](http://chess.wisc.edu/achess-archive/)

Weekly surveys through A-CHESS related to patient recent substance use, status on five protective and five risk factors from the Brief Alcohol Monitor. Information was used for triage and feedback. Case managers and counselors received a summary of each patient’s check-in data any time they wished, the day before a scheduled appointment, and whenever a patient reported a lapse.

the research team, two separate network diagrams were prepared a) participants and staff, b) a network of participants only

**A-CHESS use.** de-identified data on logins and page views were collected by computer at the Center for Health Enhancement Systems Studies, University of Wisconsin–Madison, where the app was developed.

**GPRA reports***.* intake and follow-up GPRA survey responses were entered into the federal data base by KRCC staff (PJ) and made available to author for descriptive insights.

**Outpatient treatment data from medical records.** Deidentified data: dates of birth, admission, and discharge; diagnosis; types of clinical services received; and number of service units received. Service types were group sessions, individual sessions, peer support, case management sessions, and drug screens, reported as 15-min units. Clinical service types and units were gathered for both groups post hoc. This data enabled the calculation of length of stay, number of units received of each clinical service, and total number of units received. Levene’s test was used to assess the equality of variances for retention-in-service variable distributions. This indicated inequality between the two groups and suggested non-parametric analysis. Mann-Whitney *U* and chi-square tests were used to analyze between-group effects.

**Outcome measures**

1. Muroff et al., 2017

CASA-CHESS adapted and translated A-CHESS to be linguistically and culturally relevant to Spanish-speaking Latino(a)s in recovery. Terms and phrases were added that are commonly used in Puerto Rico, Dominican Republic, and Central America. Sociocultural factors (e.g., discrimination, stigma, and acculturation) also were addressed. There are aspects of A-CHESS, providing “digital voice services, text messaging, web access, GPS, voice recognition, and video capabilities, links to resources: social support (e.g., discussion groups, personal stories), tools (e.g., relaxation exercises, appointment reminders), and information services (e.g., medication information, the effects of specific substances). A new medication adherence survey was developed specifically for CASA-CHESS that recorded adherence and/or nonadherence that alerted the case manager when medications were not taken, and linked the client to a case manager. The A-CHESS app (English only) was made public and is available on iOS and Android devices. A demonstration of A-CHESS and screenshots of CASA-CHESS are available at https:// chess.wisc.edu/achess-archive/CASA.

Hypothesis: patients who used A-CHESS would have better retention in treatment than those without it (length of time between admission and dis- charge dates). Also, they would access more care (number of units of clinical service: type and sum of all service units).

Usage data, login time stamps and services used within the CASA-CHESS app (e.g., messages, discussion board, and recovery information) were recorded. Engagement was assessed by operationalizing the number of days that the clients had access to the app, using four months as a post-residential bench- mark and as a comparison to A-CHESS. Potential usage days were adjusted for “loss episodes,” when clients did not have access to CASA-CHESS due to lost, stolen, or broken phones, time in jail, or return to residential treatment. Clients who were still actively using CASA-CHESS four months after receiving the phone were coded as “4C finishers” versus those who did not whom were coded as “discontinued prior to four months.” Page views were counted if users accessed (i.e., clicked, used, viewed) CASA-CHESS services (i.e., within-app content). Replicating A- CHESS’ visualizations, percentages of overall use included the overall participant group (N D 79) as a denominator; thus, visualizations did not account for unique daily contributions.

Univariate analyses of means (M) and standard deviations (SD) were run on demographics and app use. Bivariate analyses, including chi-square analyses and t- tests, were conducted for each

1. Shrestha et al., 2017

Outcomes: to receive medication reminders, to receive information about HIV risk reduction, and to assess HIV risk behaviors. Participants were asked three items, which were measured on a 5-point Likert scale (ranging from 1 = ‘not interested at all’ to 5 = ‘extremely interested’). These variables were further dichotomized as “No” (not interested at all) and “Yes” (slightly interested, somewhat interested, moderately interested, and extremely interested). Socio-demographic characteristics, health insurance status, visit to healthcare provider, homeless status, currently taking prescribed medication, self-perceived HIV risk, and satisfaction with current HIV prevention methods were assessed.

**HIV risk behavior scale (HRBS):** Current HIV risk behaviors Brief Inventory of Neurocognitive Impairment (BINI): measured neurocognitive impairment a 54-item self-report measure of neuropsychological symptoms

**Center for Epidemiological Studies Depression (CES-D) Scale:** assessed depressive symptoms; the 20-item **Alcohol Use Disorders Identification Test (AUDIT):** measured alcohol use disorder; validated 10-item

demographic variable with the dependent variable (4C finishers versus the discontinued groups).

Regression analyses were used to examine whether age predicted use of the app (pages viewed). Analyses were run using SPSS 24. Figures were developed in Tableau 10.0.9 and Excel 2016.

SPSS 23.0 and criterion for statistical significance was p<0.05 were used for the analysis.

Descriptive statistics such as frequencies and percentages for categorical variables, and means and standard deviations for continuous variables were taken. After conducting bivariate analyses for significant associations with the three primary outcomes, a multivariate logistic regression analyses on bivariate associations was conducted and found to be significant at p < 0.10. The final model was based on goodness-of-fit using the Hosmer and Lemeshow Test.

**References**

1. Johnson, K., Richards, S., Chih, M.-Y., Moon, T. J., Curtis, H., & Gustafson, D. H. (2016). A Pilot Test of a Mobile App for Drug Court Participants. *Substance Abuse: Research and Treatment*, *10*, SART.S33390. https://doi.org/10.4137/SART.S33390
2. Johnston, D. C., Mathews, W. D., Maus, A., & Gustafson, D. H. (2019). Using Smartphones to Improve Treatment Retention Among Impoverished Substance-Using Appalachian Women: A Naturalistic Study. *Substance Abuse: Research and Treatment*, *13*, 1178221819861377. https://doi.org/10.1177/1178221819861377
3. Lehman, W. E. K., Pankow, J., Muiruri, R., Joe, G. W., & Knight, K. (2021). An evaluation of StaySafe, a tablet app to improve health risk decision-making among people under community supervision. *Journal of Substance Abuse Treatment*, *130*, 108480. https://doi.org/10.1016/j.jsat.2021.108480
4. Muroff, J., Robinson, W., Chassler, D., López, L. M., Gaitan, E., Lundgren, L., Guauque, C., Dargon-Hart, S., Stewart, E., Dejesus, D., Johnson, K., Pe-Romashko, K., & Gustafson, D. H. (2017). Use of a Smartphone Recovery Tool for Latinos with Co-Occurring Alcohol and Other Drug Disorders and Mental Disorders. *Journal of Dual Diagnosis*, *13*(4), 280–290. https://doi.org/10.1080/15504263.2017.1348649
5. Shrestha, R., Karki, P., & Copenhaver, M. (2017). Interest in use of mHealth technology in HIV prevention and associated factors among high-risk drug users enrolled in methadone maintenance program. *AIDS Care*, *29*(9), 1144–1148. https://doi.org/10.1080/09540121.2017.1325439
