# Supplementary material for: Training an AI Chatbot to Manage Health in Underserved Populations: Methodological Approach
Source: JMIR AI. 2026 Apr 1;5:e84145. doi: 10.2196/84145 (PMC13085989; doi:10.2196/84145)
Supplement: Multimedia Appendix 3 [file ai_v5i1e84145_app3.pdf]

Appendix 3
Study 3: Intervention Development Study to Test the Feasibility, Usability, and Acceptability of mHealth Apps

Table S1: *Features of the mHealth apps*

#	mHealth App	Frequency of App Use	Features of the App
1	uSafeUS	Acutely, during times of imminent threat or violence	• Designed for college women to assist in obtaining help during times of violence, imminent harm, and resources specific to sexual/dating violence.<br>• The app developer agreed to tailor the app for our participant population with a default tab named "demo college" for our participants for study use.<br>• When participants accessed, they were able to use features such as triggering fake calls or texts to give them a reason to leave an area. Another feature was to alert trusted friends or family when participants did not get home or to their destination on time.
2	Nice Sex Tracker	Daily, as applicable	• Tracks sexual partners, locations, activities, and STDs in a discreet and nonjudgmental style.<br>• Provides analytics of sexual health and behaviors and provides resources information, where to get STDs testing.<br>• Participants were instructed to interact with app daily. There are tabs within the app to input time, activities, partners, location, and barrier methods within sexual encounters.<br>• Tracking also includes sexual intimacy activities, lab or test results, and information about partners.

Table S2: *Interview guide* using Social Cognitive Theory as the guiding framework

#	Question Type	Question & Aims
1	Personal/ Individual Factors	• Describe how you felt when using the [app name] mHealth app (i.e.: comfort, support, frustration, empowered, etc.).?<br>• What assisted you or was a barrier in using the app for the first time; from continuing to you the app?<br>• What would make the app more appealing or usable for you? (colors, fonts, representation)<br>• What do you think has to be addressed to make something like this easier for you to use? Such as training on how to use the app?
1	Behavioral Factors	• Were you motivated to use the app? What motivated you to use the mHealth app such as a desire to be in control of your health, your role as a caregiver, mother, hope for the future etc.?<br>• If you were not motivated to use the app, can you describe the reasons such as: inconvenience, not interested, do not like technology etc.?
2	Environmental Factors	• Describe what it was like using the [name of app] within your environment such as at home, at work, in the community (park, vehicle, on the street, in waiting rooms, appointments, etc).<br>• What assisted or became a barrier to using the app in these places?<br>• What do you think has to be addressed to make something like this easier for you to use? Such as assistance with cell phone plans, restrictions on data sharing/mandatory reporting; training for agencies who use this?<br>• What concerns do you have with people or systems (PO, probation, counselors, HCPs) having access to the app, your data, and knowing you use this app?<br>• Are there features or data you should have access to while having other pieces of the data be restricted?
3	Personal/ Individual Factors<br>Behavioral Factors	• Tell me how using the [name of app] improved how you felt about managing your health and safety?<br>• Was there anything within the app that decreased your feelings of being capable of managing your health and safety?<br>• Which health and safety features did you find most/least useful?<br>• How did your health/safety improve or decline while using the app?
4	Personal/Individual Factors	• Can you describe what ways your knowledge about your sexual health and safety increased while using [name of app]?<br>• What personal or environmental factors helped with increasing your knowledge such as previous experience using mHealth apps; prior experience managing these health issues; training opportunities; systems allowing you to use the app etc.?

5	Personal/Individual Factors	• How did the use of [name of app] help manage your daily/ emergency/ acute sexual health behaviors? Such as behaviors in safe sex practices; increasing agency and autonomy during sexual encounters; tracking symptoms of STI/STD; tracking; reporting; or identifying instances of imminent or experienced sexual violence?<br>• What outcomes would you like more assistance with and that are most important to you?
6	Personal/Individual Factors	• How did the use of [name of app] assist in accessing resources or support specific to your sexual health and safety?<br>• What concerned you about your confidentiality with the legal, court, CPS, or MAT system, with your partner or family?<br>• How was your health improved or restricted when accessing these resources and support system?

Table S3: *Length of abuse/violence and reasons for not reporting*

#	Length of time of the abuse	Reason for not reporting
1	20-30 times	Scared, last time was 4 years ago while on probation.
2	2 times	Scared of more violence. It has affected her mental health till this day.
3	3 times	She tried, but the perpetrator was her mom's boyfriend. When she told her family, nobody believed her. She was on parole and her parole officer was the only one that believed her and helped her report it. The courts decided since his penis was "flaccid", it wasn't considered an assault.
4	1 time	It was during her substance use disorder and she figured nobody would take her seriously.
5	15 years	Fear of retaliation, CPS involvement, fear for the safety of her children, and shame from family and friends
6	Throughout childhood	Fear of retaliation from family. Her dad, the perpetrator was a preacher
7	1	Was in jail at the time

Table S4: *Principles of SCT and Exemplars*

Principal of SCT	Exemplar Describing the Principle	Definition of how these factors influence self-efficacy and outcomes
Personal Factors	*It made me take time for myself*	• SCT predicts that goals can be motivating factors for self-efficacy and behavior change.<br>• Goals help individuals focus efforts at a task and by allowing the opportunity to observe and evaluate one's progress, will lead people to be persistent to their desired goal.
Behavioral Factors	*It helped me to be more respectful of my body*	• Behavioral factors that motivate outcomes and self-efficacy are having the ability to have agency in activity, effort, persistence, achievement, and environmental regulation.
Environmental Factors	*The connectivity…. that was helpful*	• Environmental factors such as programming, accessibility to interventions, peer support, and feedback, can affect an individual's outcomes and self-efficacy.
