# Supplementary material for: Training an AI Chatbot to Manage Health in Underserved Populations: Methodological Approach
Source: JMIR AI. 2026 Apr 1;5:e84145. doi: 10.2196/84145 (PMC13085989; doi:10.2196/84145)
Supplement: Multimedia Appendix 4 [file ai_v5i1e84145_app4.pdf]

Appendix 4

Rigor Cycle Study 1: Exploring JUN™ Amongst Women with Histories of Community Supervision

Table S1: *Rigor Cycle Study 2 Exploring JUN™ Social Cognitive Theory Interview Guide*

#	Question Type	Question & Aims	Probe(s)/follow-up
1	Personal/ Individual Factors [A]	Describe how you felt when using JUN?	Tell me more
	EPIS barriers and facilitators	What assisted you or was a barrier in using the app for the first time; from continuing to you the app?<br><br>What would make the app more appealing or usable for you? (colors, fonts, representation)	How were the daily features compared to the emergency or acute features within the app?
2	Behavioral Factors [B]	Were you motivated to use the app? What motivated you to use the mHealth app such as a desire	What suggestions do you have to tailor it to your needs

		to be in control of your health, your role as a caregiver, mother, hope for the future etc.?<br><br>If you were not motivated to use the app, can you describe the reasons such as: inconvenience, no interested, do not like technology etc.?	such as prompts, language, design, timing of reminders, types of reminders, confidentiality issues?
3	Environmental Factors [C]<br><br>EPIS barriers and facilitators	Describe what it was like using JUN within your environment such as at home, at work, in the community (park, vehicle, on the street, in waiting rooms, appointments, etc.<br><br>What assisted or became a barrier to using the app in these places?<br><br>Are there features or data you should have access to while having other pieces of the data be restricted?	Was using the app better in daily use or instances of emergency that was best in helping you get support/resources?<br><br>What ways can we tailor or improve these features?<br><br>Can we add anything more?
4	Personal/Individual Factors [A]	Tell me how using JUN improved how you felt about managing your health and safety?<br><br>Which features did you find most/least useful?	What did you not like?
5	Knowledge [b]	Can you describe what ways your knowledge about your health increased while using JUN?<br><br>What personal or environmental factors helped with increasing your knowledge such as previous experience using mHealth apps; prior experience managing these health issues; training opportunities; systems allowing you to use the app etc.?	
6	Self-management behavior [c]<br><br>Personal/Individual Factors [A]	How did the use of JUN help manage your daily or acute health behaviors (if applicable).	
