# Supplementary material for: Training an AI Chatbot to Manage Health in Underserved Populations: Methodological Approach
Source: JMIR AI. 2026 Apr 1;5:e84145. doi: 10.2196/84145 (PMC13085989; doi:10.2196/84145)
Supplement: Multimedia Appendix 5 [file ai_v5i1e84145_app5.pdf]

Appendix 5

Rigor Cycle Study 2: Testing JUN™ Amongst Pregnant Women

Table S1: *Health Belief Model Interview Amongst Pregnant Women with and without Community Supervision*

Question	Probe Question
Describe your experience with this pregnancy.	What are your feelings Happy, sad, scared etc.
How was your life before getting pregnant?	Demographics: marital status, health status, support, employment, education, housing, etc.)
What is your life now being pregnant? Have things changed?	
How do you view your overall health?	
If you need to get healthcare, how do you go about getting care?	
Describe the health symptoms you have experienced so far.	
How do you prioritize your health? Has it changed during pregnancy?	
What are barriers to managing your health or seeking health care when needed?	
What do you think are the benefits to managing your health?	
Has the way you managed your health changed after using JUN? How so?	
