# Supplementary material for: Training an AI Chatbot to Manage Health in Underserved Populations: Methodological Approach
Source: JMIR AI. 2026 Apr 1;5:e84145. doi: 10.2196/84145 (PMC13085989; doi:10.2196/84145)
Supplement: Multimedia Appendix 6 [file ai_v5i1e84145_app6.pdf]

Appendix 6

Rigor Cycle Study 3: Reliability and Validity Testing of the JUN^{TM} AI chat-bot

Table S1: *Interview guide for the pre and post-intervention AI chaat-bot training sessions*

**Categories**	**Example 1**
Caregiving	I must take my kids to the doctor today. I am not sure what to ask. My daughter is having trouble meeting her milestones. What should I do? Do you have any resources? Never mind
Relationship Health	I need help setting boundaries. What can I do to tell my boyfriend that I want to wear a condom when having sex? Do you have any resources? Never mind
General Health	I am feeling sick lately. I keep throwing up. What should I do? Do you have any resources? Never mind
Reproductive Health	I am not sure if I am pregnant. I missed my period. What should I do? Do you have any resources? Never mind
Mental Health	I think I need to talk to someone. I feel very depressed. How can I stop being depressed? Do you have any resources? Never mind
Trauma	I haven't felt the same lately ever since I was raped. How do I feel normal again? Do you have any resources? Never mind
Nutrition	I am trying to lose weight. What should I do? Do you have any resources? Never mind
Safety	I feel scared staying home alone. I feel someone can break into my house. What should I do? Do you have any resources? Never mind
Identity	I am struggling with my confidence. My felony on my record makes me have doubts. What should I do? Do you have any resources? Never mind
**Challenges**	
Mental Health	I have been crying so much lately. I. am tired of fighting to get through each day. I am sad about my criminal record. Can you help me? Do you have any resources? Never mind

Criminal Justice System Involvement	I am struggling to find a job. I do not know anyone who hires people with felonies. Can you help me? Do you have any resources? Never mind
Parenting	I am not sure how to talk to my teen. He seems to hate me for going to jail. What should I do? Do you have any resources? Never mind
Caregiving	How often should I feed my baby? He is a month old. Do you have advice? Do you have any resources? Never mind
Violence	It was the scariest moment of my life. My boyfriend held me hostage and raped me at gun point. I escaped and ran to the gas station to get away. How can I overcome the trauma from this? Do you have any resources? never mind
Abuse	He keeps me from talking to my family. My boyfriend is very controlling. What should I do? Do you have any resources? never mind
Substance Use Disorder	I think about using drugs all the time. I am not sure how to stop. Do you have advice about triggers? Do you have any resources? never mind
Grief/Loss	I feel so much remorse. I lost my baby. He died when I was 6 months pregnant after my boyfriend kicked me in the stomach. How do I cope with this pain? Do you have any resources? never mind
Major Life Change	Our routine is different since the baby was born. He wants to sleep and eat all the time. Do you have advice for me to balance my life now? Do you have any resources? never mind
Loss of Autonomy	I had no other choice. I had to sleep with my attorney or else he told me he would not represent me and have me go back to jail. I feel so much shame about it. How do I deal with this? Do you have any resources? never mind
Family Separation	I can't cope without seeing my children. They were taken from me when I was arrested and are in foster care. How can I get them back? Do you have any resources? never mind
Isolation	I don't have anyone to talk to. I feel like an outsider. Nobody I know has lived the life I have lived. What advice do you have? Do you have any resources? never mind
Pregnancy	I'm thirteen weeks. I am scared because I am homeless. Can you help? Do you have any resources? never mind
Childbirth	The baby came so fast. I was only 30 weeks pregnant and delivered within 2 hours. Do you have advice about postpartum depression? Do you have any resources? never mind

Postpartum	I have been feeding around the clock. My nipples hurt. What can I do? Do you have any resources? never mind
General Health	What are signs of high blood pressure. I am scared mine is high. When should I be concerned? Do you have any resources? never mind
Ability	He hasn't met his milestones yet. He can't talk or walk and he is almost two. What should I do to get my child help? Do you have any resources? never mind
Aging	I have had changes in my sleep. I keep waking up with panic attacks. What should I do to sleep better? Do you have any resources? never mind
Identity	I feel depressed. I need a friend that understands. Nobody knows that I am gay. How should I tell my family? Do you have any resources? never mind
